# Development of a Real-Time PCR Assay for the Detection of the Phlebovirus Fermo Virus

**DOI:** 10.3390/v15102082

**Published:** 2023-10-12

**Authors:** Mattia Calzolari, Simone Russo, Katia Marzani, Gastone Dalmonte, Matteo Ricchi, Paolo Bonilauri

**Affiliations:** Istituto Zooprofilattico Sperimentale della Lombardia e dell’Emilia Romagna “B. Ubertini”, 25124 Brescia, Italy; simone.russo@izsler.it (S.R.); katia.marzani@izsler.it (K.M.); gastone.dalmonte@izsler.it (G.D.); matteo.ricchi@izsler.it (M.R.); paolo.bonilauri@izsler.it (P.B.)

**Keywords:** Fermo virus, sand fly, real-time PCR, digital PCR

## Abstract

Fermo virus is a *Phlebovirus* that is increasingly reported in sand flies from northern Italy. The natural cycle is not fully understood, but the virus has been detected by direct methods only in sand flies. Although there is serological evidence that it can infect vertebrates, the virus has not been directly detected in animals or humans. Here, we have developed and reported a specific real-time PCR for Fermo virus. The availability of the described method will be useful to characterize the epidemiology of the FERV, ensuring, compared to previously available protocols, a more sensitive detection in insects and the possible detection in vertebrates to evaluate the presence of reservoirs and the pathogenic potential of the virus in humans or animals.

## 1. Introduction

The Fermo virus (FERV), genus *Phlebovirus*, family *Phenuiviridae*, order *Bunyavirales,* is a virus characterized by a linear negative-stranded RNA genome encoding six proteins; the genome is composed of large (L, 6.4 kb), medium (M, 3.2 kb) and small (S, 1.7 kb) segments, with the S segment characterized by an ambisense transcription strategy [1]. The L segment encodes the viral polymerase (transcriptase), the M segment encodes two envelope glycoproteins (Gn and Gc), and the S segment encodes the nucleoprotein and non-structural proteins [2].

Recently, an increasing number of phleboviruses have been isolated or detected, mainly in the Mediterranean region [3]. The genus *Phlebovirus* includes several viruses mainly isolated and detected in sand flies, although the prototype of the genus, Rift Valley fever virus (RVFV), is an arbovirus mainly transmitted by mosquitoes, and several of these viruses have only been detected in vertebrates [4]. In addition to RVFV, this group includes pathogenic viruses such as Toscana virus, which causes febrile forms, meningitis and meningoencephalitis in the most serious cases; Sandfly fever Naples and Sicilian viruses and several viruses from South America can cause self-limiting febrile forms [5].

Fermo virus was firstly isolated in 2012 in the Marche region (Italy) from pools of sand flies *Phlebotomus perfiliewi* [6]. The virus has been detected and isolated in the neighboring regions of Emilia-Romagna and Lombardy [7] and shows a relevant identity to the partial sequences of Balkan virus, a virus detected in Balkan countries [8]. Phylogenetic analysis places FERV in the Naples phlebovirus group, which also includes the Toscana virus [9]. According to the ICTV species demarcation criterion—less than 95% identity in the amino acid sequence of the RNA-directed RNA polymerase with respect to other phleboviruses—FERV belongs to an independent species [9]. Serological studies carried out in domestic animals in Emilia-Romagna highlighted the potential of FERV to infect vertebrates, particularly sheep and goats [10]. Despite these observations, the virus cycle remains largely uncharacterized, as does its potential pathogenicity.

For diagnosis, the development of a real-time PCR (RT-PCR) is useful for the detection, quantification and typing of *Phlebovirus*. The use of culture methods for the detection of important clinical and veterinary viruses is difficult or impossible. Serological tests such as ELISA are characterized by a relatively low sensitivity and specificity. On the other hand, RT-PCR is a fundamental assay for viral diagnostics, not only because of its good sensitivity and specificity, but also because of its ability to determine the viral load and, consequently, the efficiency of the antiviral therapies [11].

The aim of this study is to develop a specific RT-PCR for the detection of FERV to allow for a more sensitive detection in insects and its potential detection in vertebrates. In our study, we evaluated the analytical sensitivity (LOD_95%_) and the diagnostic performance of the RT-PCR and also built standard curves for RNA quantification using positive controls quantified by digital PCR (dPCR).

## 2. Material and Methods

### 2.1. Primers and Probe Definition

**Figure 1:**
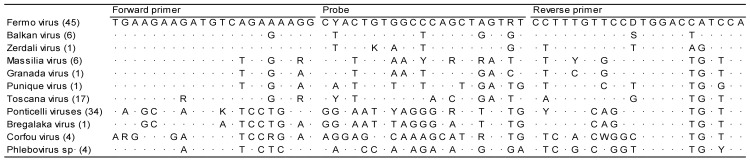
Primers and probe sequences recorded in Fermo virus and in homologous sequences of other phleboviruses deposited in GenBank. GenBank accession numbers: Fermo virus: KY354388, KY354389, KY354390, KY354391, KY354392, KY354393, KY354394, MG869823, MG869824, MG869827, MG869828, MG869830, MG869833, MG869835, MG869836, MG869837, MG869838, MG869839, MG869841, MG869845, MG869846, MG869847, MG869851, MG869852, MG869855, MG869859, MG869861, MG869870, MG869871, MG869872, MG869880, MG869881, MG869882, MG869883, MG869884, MG869885, MG869886, MG869887, MG869892, MG869893, MG869894, OP485761, OP485762, OP485763, OU230767; Balkan virus: KY662276, KY662277, KY662278, KY662280, KY662281, KY662282; Zerdali virus: NC_037613; Massilia virus: KT783485, KT783486, KT906102, KT906102, KT906103, NC055415; Granada virus: GU135608; Punique virus: NC055300; Toscana virus: KM275237, KM275764, KM275768, KM275772, KM275776, KM275778, KM275779, KM275780, KM275784, KM275785, KM275787, KU935738, MG869826, MG869832, MG869840, MG869895, MN940423; Ponticelli viruses: KX388213, KX388216, KX388219, KX388222, KX388225, KY354371, KY354373, KY354374, KY354375, KY354376, KY354377, KY354378, KY354379, KY354380, KY354381, KY354382, KY354383, KY354384, KY354385, KY354387, MG869825, MG869834, MG869844, MG869869, MG869873, MG869874, MG911975, MG911980, MG911983, MG911986, MG911989, MH427535, MH427536, OP293793; Bregalaka virus: MG573146; Corfou virus: EF201821, KR106179, MG869875, MG869889; Phlebovirus sp.: MG869843, MG869866, MG869868, MG869891.

Several deposited sequences of FERV refer to the application of the pan-phlebovirus PCR (Phlebo-PCR) proposed by Lambert and Lanciotti (2009), which amplifies a conserved tract of the S segment of phleboviruses. Using this tract, FERV sequences and homologous sequences of other phleboviruses were retrieved from GenBank, paying particular attention to phleboviruses similar to FERV and phleboviruses detected in the Emilia-Romagna region. Sequences were aligned using MAFFT [12], and primers and probes were designed using only those parts conserved in FERV (Figure 1). We define the forward primer as F-FER: 5′-TGA AGA AGA TGT CAG AAA AGG G-3′, the reverse primer as R-FER: 5′-TGG ATG GTC CAT GGA ACA AAG G-3′, and the probe as FER-S: 5′-FAM CYA CTG TGG CCC AGC TAG TRT C 3′-BHQ1; the target amplicon is 136 bases long.

### 2.2. Standard Curve Building

#### 2.2.1. Viral RNA Extraction

The standard curve was built using tenfold dilutions of a positive control obtained from a strain of FERV isolated from IZSLER in 2018 (212236/3 isolated), grown on VERO cells (7th passage). The cultured virus was extracted using the KingFisher™ Flex Purification System (Thermo Fisher Scientific, Milan, Italy) according to the manufacturer’s instructions. The concentration of viral RNA was then quantified.

#### 2.2.2. RNA Quantification of the Standards

The 50% Tissue Culture Infectious Dose (TCD50) was determined in VERO cells (7th passage) and was 10^4.9^ TCID50/50 µL. The standard was further quantified by digital PCR (dPCR). Briefly, the extract obtained was diluted tenfold to 10^−5^ and quantified by dPCR (QIAcuity ONE Digital PCR System, Qiagen, Milan, Italy). A 24-well plate with 8500 partitions was used for this quantification. The reaction mix consisted of 3 μL of a 4X QuantiTECT ^®^ Virus + ROX mix (Qiagen), 1.55 μL of a primer and probe mix (50 µL of 100 µM primers, 35 µL of 100 µM probe and 365 µL of sterile water), 0.12 μL of a 100X reverse transcriptase and 1.33 µL of sterile water, per reaction. For each sample, the final volume was 12 µL, 6 µL of master mix and 6 µL of RNA. The thermal profile used was that reported for the RT-PCR.

The results were provided by the QIAcuity Software Suite 2.7.7.182, which returned the average value of the sample while taking into account the generated partitions. Each value, expressed in copies/µL, was multiplied by a dilution factor of 2, corresponding to the ratio between the total volume of the RNA mix analyzed (12 µL) and the volume of RNA added (6 µL).

#### 2.2.3. Real-Time PCR Protocol and Standard Curve Standardization

The RT-PCR protocol was applied to tenfold dilutions of the viral extract (from undiluted to 10^−9^). The RNA was first reverse-transcribed using 200 U/µL Super Script II™ Reverse Transcriptase (Invitrogen by Thermo Fisher Scientific) at 42 °C for 50 min. For each reaction, 20 μL of reaction mix was obtained by mixing 4 µL of 5 × QuantiFast^®^ Pathogen Master Mix (Qiagen), 2 µM of each primer, 0.7 µM of probe, 3 µL of cDNA and distilled water. After an initial denaturation step at 95 °C for 8 min, 50 cycles were performed with the thermal profile of 94 °C for 10 s, 58 °C for 20 s and 72 °C for 30 s. The RT-PCR was performed on the CFX96 Real-Time System (Bio-Rad, Milan, Italy), and the results were analyzed using the CFX Manager Industrial Diagnostic Editor (Bio-Rad).

The standard curve was built by plotting the concentration of the dilutions of the viral control RNA against the cycle of quantification value (Cq) obtained by analyzing the dilutions by RT-PCR. We also calculated the slope, the R^2^, the intercept and the efficiency of the assay using the formula:

E = 10^−1/slope^ − 1

### 2.3. Analytical Sensitivity

To evaluate the analytical sensitivity (LOD_95%_) of the protocol, defined as the minimum amount of virus at which all the replicates are positive, we performed 10 repetitions of the RT-PCR on the positive control diluted at 10^−4^, 10^−5^ and 10^−6^. The LOD_95%_ was also evaluated by another approach, *logit* regression, using SPSS software (version 27, IBM). When analyzing the binomial response variables (positive and negative), *logit* regression transforms the sigmoid dose-response curve typical of a binomial variable into a straight line that can be analyzed by regression using either the least squares or maximum likelihood methods [13]. The result of the analysis provides the probability (e.g., 95%) of detecting the nucleic acid [14] at a given RNA concentration.

### 2.4. Diagnostic Performance

To evaluate the diagnostic performance of the method, the RT-PCR protocol was then applied to 20 pools of sand flies collected in 2021. The pools were extracted and retro-transcribed using the same protocol as previously described and were tested with the Phlebo-PCR [15] followed by sequencing and with a specific TOSV PCR [16]. The pools were classified as positive for FERV (9) or Toscana virus (4) or negative for both viruses (7) (Table 1). Diagnostic accuracy, sensitivity and specificity were evaluated according to Part B of the World Organization for Animal Health (WOAH, former OIE) (2016) [17]. The confidence interval was determined using the exact binomial test run in RStudio (RStudio version 1.4.1106).

## 3. Results

### 3.1. Standard Curve Building

**Figure 2:**
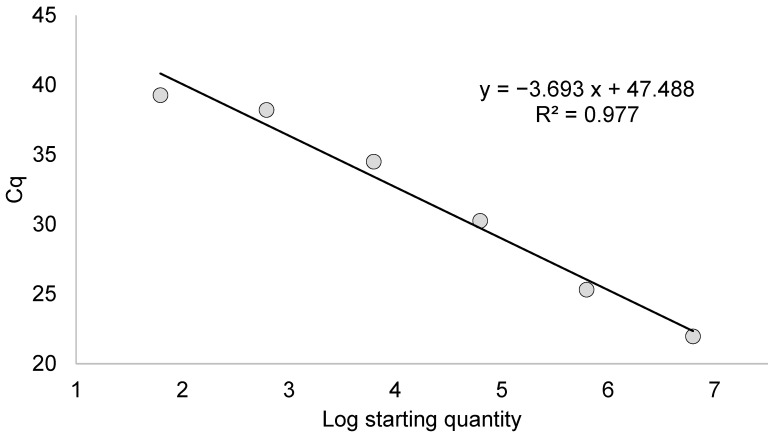
Model calibration curve with reaction efficiency, regression coefficient (R^2^), slope, and y intercept.

Quantification by dPCR using three dilutions of the positive control provided a copy count of 6260 copies/μL for 10^−3^, 616 copies/μL for 10^−4^, and 63 copies/μL for 10^−5^. Figure 2 shows the model calibration curve built by plotting the concentration of the positive viral samples on a logarithmic scale and the Cq obtained by analyzing the dilutions by RT-PCR.

The reaction efficiency was 86.6% (slope of −3.693, intercept of 47.448 and R^2^ of 0.977).

### 3.2. Analytical Sensitivity

**Figure 3:**
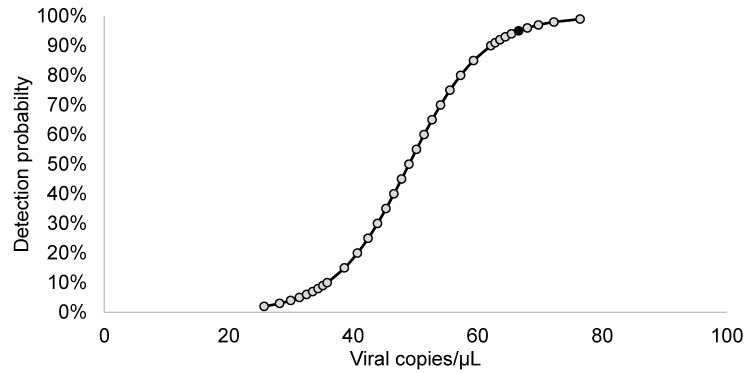
Curve obtained by the *logit* function applied to low-concentration dilutions to find the lower limit of detection of the protocol (black dot).

The LOD_95%_ of the RT-PCR was evaluated by analyzing 10 replicates of 3 virus dilutions (from 10^−4^ to 10^−6^) containing from 616 copies/μL to 6.2 copies/μL, according to dPCR. No detection was obtained for the 10^−6^ replicates, while 9 replicates were detected at 10^−5^ (average Cq value of 39.33), and all 10^−4^ replicates provided an amplification curve (average Cq value of 37.17); according to the *logit* function (Figure 3), the LOD_95%_ of the method corresponds to 66.6 copies/μL.

### 3.3. Diagnostic Performance

The RT-PCR applied on 20 pools of sand flies (9 positive for FERV, 4 positive for Toscana virus and 7 negative for both viruses) was able to detect the virus in FERV pools, while no amplification curves were obtained for TOSV-positive pools or negative pools, highlighting a good diagnostic sensitivity and specificity of the described method.

Using data obtained from the 20 available samples, the diagnostic accuracy was 100% (83–100%), while the diagnostic sensitivity and specificity were 100% (75–100%) and 100% (59–100%), respectively.

## 4. Discussion

This study reports on the development of a quantitative RT-PCR assay for the identification and quantification of the S segment of FERV.

Prior to the establishment of this protocol, other studies were published on the use of PCR for the detection of phleboviruses. In this context, Lambert and Lanciotti developed a Phlebo-PCR followed by the sequencing of the obtained amplicon [15]. Another study reported the results of a nested PCR for the detection of L and S segments of sand-fly-borne phleboviruses [18]. An RT-PCR assay using degenerate primers complementary to the L segment was performed for the detection of both tick-borne and sand-fly/mosquito-borne phleboviruses [19].

To our knowledge, the detection of FERV has been based on these protocols or on more complex and often less sensitive methods, such as whole genome sequencing techniques or virus isolation. We carried out this study for the specific detection of FERV using RT-PCR, which is able to provide both the presence and, when used with appropriate standards, the quantification of FERV. The RT-PCR showed good performance in terms of sensitivity and specificity. The LOD_95%_ calculated by *logit* regression corresponds to 66.6 copies/µL, similar to the values obtained in another study carried out on different phleboviruses [20]. The value obtained by *logit* regression was lower than that obtained by the conservative approach, where the LOD_95%_ was 616 copies/µL, considering the dilution at which all replicates were positive. However, WOAH includes the use of *probit/logit* regression in diagnostics in its documents for the validation of diagnostic assays [21].

The RT-PCR allows for the quantification of microorganisms by building calibration curves, using dilutions of standards with known concentrations or copy numbers (Bustin et al., 2009). The need for standards with a known amount of target is one of the few disadvantages of RT-PCR, but on the other hand it allows not only for the detection of the specific target, but also for its quantification over a wide dynamic range [22]. However, unknown or poorly known etiological agents, such as some phleboviruses, are increasingly detectable and characterizable only by molecular tools [5]. On the other hand, the use of dPCR, which has a low dynamic range due to saturation, allows for the rapid quantification of standards with minimal assay optimization, provided that the standards are properly diluted.

The calculation of the RT-PCR amplification efficiency using the standard curve is an important point for the quality of the method, as reported in the MIQE guidelines [22,23]. In our RT-PCR method, the efficiency is 86.6%, which is close to the optimal value of 100%. However, the RT-PCR efficiency is rarely 100%, and it usually deviates from this value.

This protocol will allow for a more sensitive detection of FERV compared to the methods previously used in insects, allowing for a more accurate assessment of the temporal and spatial dynamics of the virus. We recognize that only a few samples have been used to determine accuracy, specificity and sensitivity, so the inclusion of more samples and the inclusion of other phleboviruses could be pivotal for a better determination of these parameters. A further effort to establish this protocol on vertebrate samples will allow one to evaluate the infectivity and potential pathogenicity of FERV for animals and humans.

In any case, we suggest that the use of this protocol will be a useful tool to clarify the life cycle of FERV.

## Figures and Tables

**Table 1:** Details of sampling of sand fly pools tested with the proposed protocol with reference to detection of Fermo virus (FERV) and Toscana virus (TOSV).

Day	Municipality	N	FERV	TOSV PCR	FERV RT-PCR
10 August 2021	Serravalle (BO)	100	p *		p
10 August 2021	Serravalle (BO)	100	p *		p
10 August 2021	Serravalle (BO)	100	p *		p
24 August 2021	Serravalle (BO)	100	p *		p
27 August 2021	Sadurano (FC)	50	p *		p
27 August 2021	Sadurano (FC)	50	p *		p
27 August 2021	Sadurano (FC)	49	p *		p
19 August 2021	Cesena (FC)	20	p *		p
10 August 2021	Budrio (BO)	24	p *		p
29 September 2021	Pianoro (BO)	69		p	
7 September 2021	Monteveglio (BO)	24		p	
24 August 2021	Serravalle (BO)	100		p	
10 August 2021	Serravalle (BO)	100		p	
7 September 2021	Vignola (MO)	100			
7 September 2021	Vignola (MO)	100			
7 September 2021	Vignola (MO)	100			
7 September 2021	Vignola (MO)	100			
7 September 2021	Vignola (MO)	100			
7 September 2021	Vignola (MO)	100			
7 September 2021	Vignola (MO)	100			

* Obtained by sequencing of application of Pan-phlebovirus PCR according to Lambert and Lanciotti, 2009 [15].

## Data Availability

The data presented in this study are available in the article.
